# Supplementary figures and images for: Reversible translocation of acyl-CoA:cholesterol acyltransferase (ACAT) between the endoplasmic reticulum and vesicular structures
Source: Front Mol Biosci. 2023 Nov 10;10:1258799. doi: 10.3389/fmolb.2023.1258799 (PMC10667705; doi:10.3389/fmolb.2023.1258799)

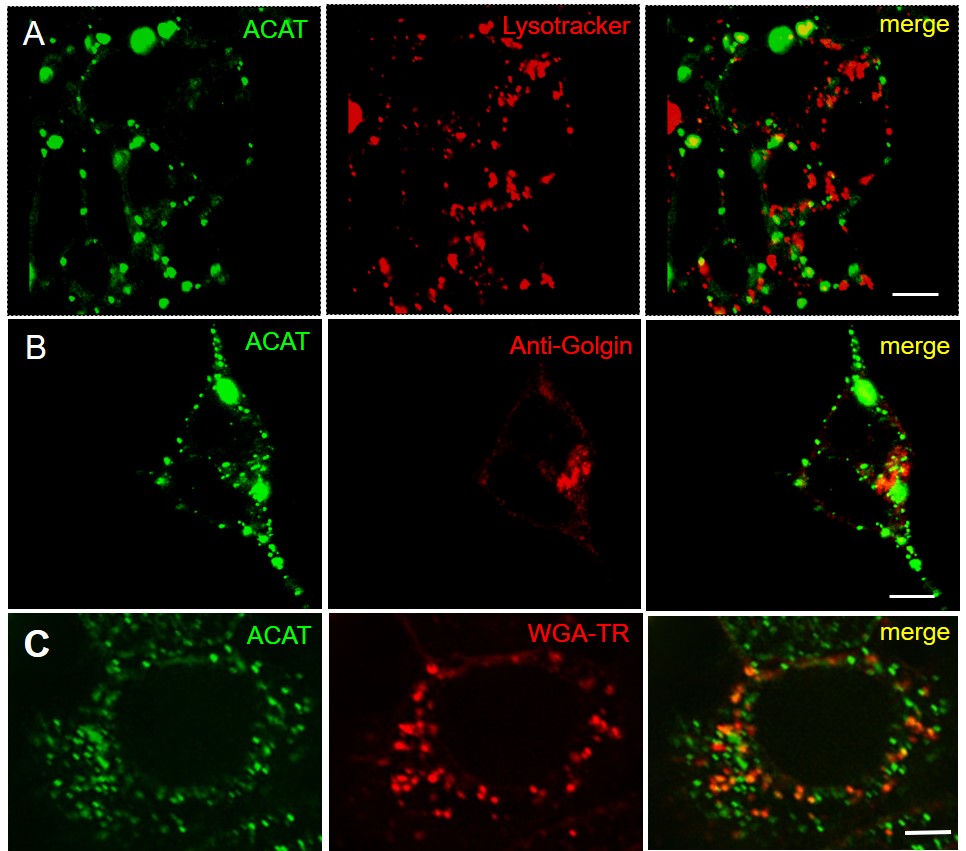

Supplement: Supplementary file 1 [file Image3.JPEG]

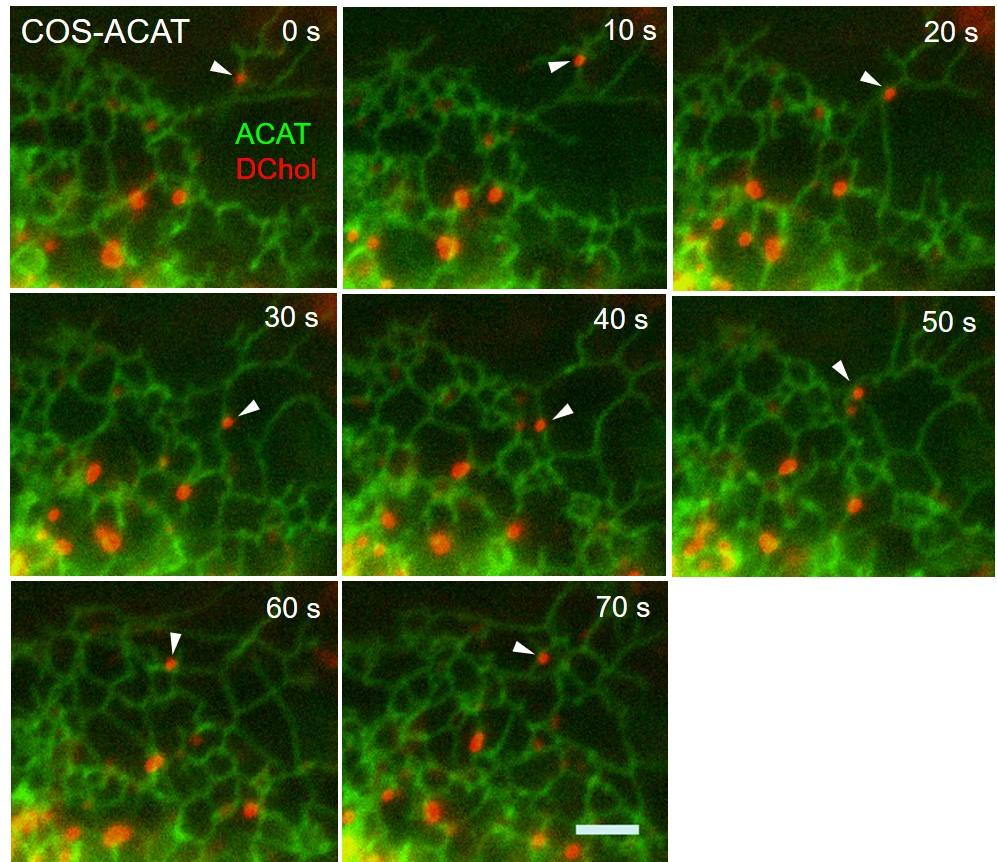

Supplement: Supplementary file 2 [file Image1.JPEG]

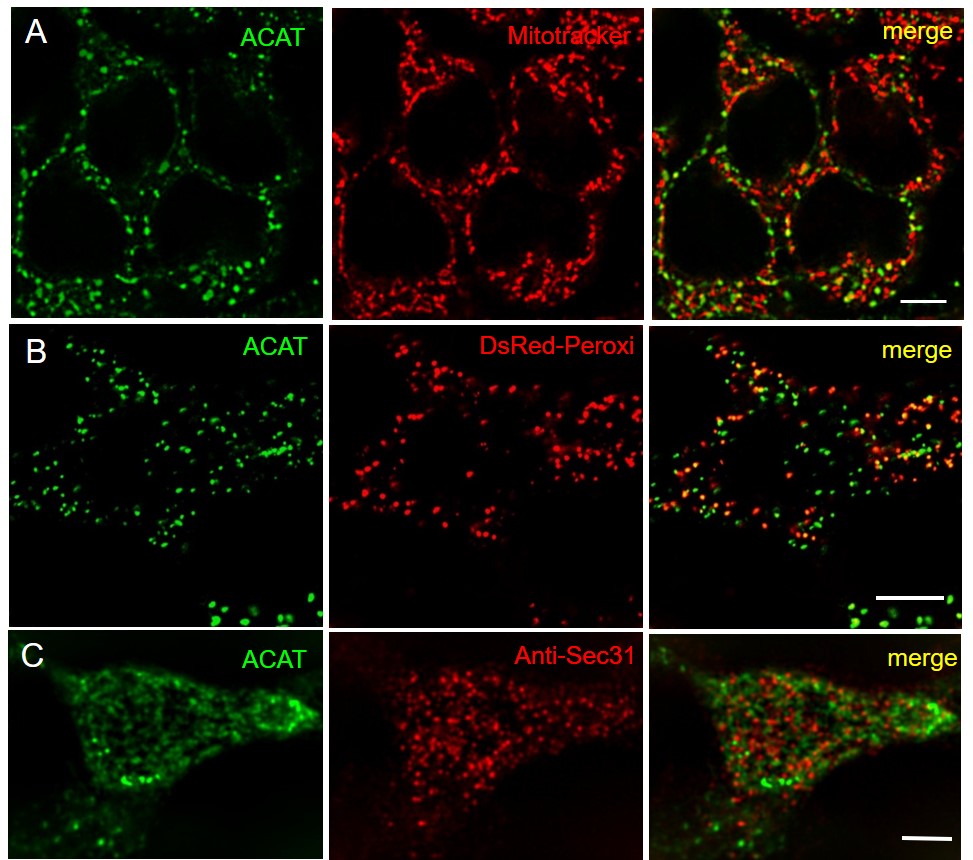

Supplement: Supplementary file 3 [file Image4.JPEG]

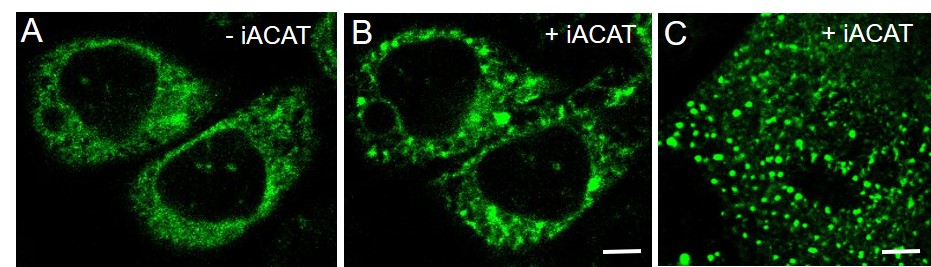

Supplement: Supplementary file 4 [file Image2.JPEG]
